# Nitrous Oxide Production in Co- Versus Counter-Diffusion Nitrifying Biofilms

**DOI:** 10.1038/srep28880

**Published:** 2016-06-29

**Authors:** Lai Peng, Jing Sun, Yiwen Liu, Xiaohu Dai, Bing-Jie Ni

**Affiliations:** 1 State Key Laboratory of Pollution Control and Resources Reuse, College of Environmental Science and Engineering, Tongji University, Shanghai 200092, PR China; 2 Laboratory of Microbial Ecology and Technology (LabMET), Ghent University, Coupure Links 653, Ghent 9000, Belgium; 3 Centre for Technology in Water and Wastewater, School of Civil and Environmental Engineering, University of Technology Sydney, Sydney, 2007 NSW, Australia

## Abstract

For the application of biofilm processes, a better understanding of nitrous oxide (N_2_O) formation within the biofilm is essential for design and operation of biofilm reactors with minimized N_2_O emissions. In this work, a previously established N_2_O model incorporating both ammonia oxidizing bacteria (AOB) denitrification and hydroxylamine (NH_2_OH) oxidation pathways is applied in two structurally different biofilm systems to assess the effects of co- and counter-diffusion on N_2_O production. It is demonstrated that the diffusion of NH_2_OH and oxygen within both types of biofilms would form an anoxic layer with the presence of NH_2_OH and nitrite ( 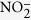), which would result in a high N_2_O production via AOB denitrification pathway. As a result, AOB denitrification pathway is dominant over NH_2_OH oxidation pathway within the co- and counter-diffusion biofilms. In comparison, the co-diffusion biofilm may generate substantially higher N_2_O than the counter-diffusion biofilm due to the higher accumulation of NH_2_OH in co-diffusion biofilm, especially under the condition of high-strength ammonium influent (500 mg N/L), thick biofilm depth (300 μm) and moderate oxygen loading (~1–~4 m^3^/d). The effect of co- and counter-diffusion on N_2_O production from the AOB biofilm is minimal when treating low-strength nitrogenous wastewater.

The destruction of stratospheric ozone layer has become a significant environmental issue in 21^st^ century, which is contributed largely by nitrous oxide (N_2_O)^1^. According to IPCC^2^, the N_2_O concentration in global atmosphere has increased from pre-industrial value of around 271 ppbv to 324 ppbv in 2011 due to human activities comprising agriculture, industry, fossil fuel combustion, and wastewater treatment. N_2_O is also a potent greenhouse gas with a global warming potential (GWP) of approximately 265 times stronger than carbon dioxide^2^. In terms of this, even a low emission of N_2_O from wastewater treatment is environmental hazardous and thus unwanted.

Ammonia oxidizing bacteria (AOB) have been recognized as the main contributor to N_2_O production from wastewater treatment plants (WWTPs)^3^,^4^,^5^,^6^. It is commonly believed that N_2_O is generated by AOB via two pathways: the first involves sequential reductions from nitrite (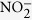) to nitric oxide (NO) and from NO to N_2_O as the end product, termed as AOB denitrification pathway^4^,^7^,^8^,^9^; the second includes incomplete oxidation of hydroxylamine (NH_2_OH) with N_2_O as the side product, known as NH_2_OH oxidation pathway^8^,^10^,^11^. The AOB denitrification pathway is favored at oxygen (O_2_) limiting conditions with nitrite accumulation^4^,^7^,^12^, whilst the NH_2_OH oxidation pathway is significantly promoted by elevated oxygen concentration^8^,^13^. Therefore, the varying DO and nitrite concentrations may trigger the shift of the two known N_2_O pathways^6^,^14^.

To reveal the mechanism of N_2_O production by AOB and enhance our ability to predict site-specific N_2_O production in WWTPs, Ni *et al*.^15^ developed a integrated N_2_O model incorporating both AOB denitrification and NH_2_OH oxidation pathways. Electron mediators (Mred as the reduced form and Mox as the oxidized form) were used to model the electron transfer from oxidation to reduction in biochemical reactions. This two-pathway model has been evaluated using the experimental data from several highly different cultures performing nitritation and/or nitrification, respectively and successfully applied for prediction of N_2_O production from a step-feed full-scale wastewater treatment reactor^15^,^16^.

There is a rapid development and growing utilization of biofilm processes (e.g. membrane biofilm reactors, fluidized bed reactors, rotating biological contractors, granular biofilter, trickling filters etc.) during wastewater treatment. Different from the suspended-growth sludge, N_2_O production from the biofilm may be affected by the stratification of electron donors, acceptors and microbes. For example, based on mathematical modeling, Sabba *et al*.^17^ observed that the diffusion of NH_2_OH from aerobic region to anoxic region would result in a substantially higher N_2_O production in biofilm than that in suspended-growth sludge.

In conventional biofilm systems, all of the substrates are supplied into the biofilm from the bulk liquid, while in membrane aerated biofilm reactors (MABR), the oxygen and other substrates are dividedly provided to the biofilm base through a gas-permeable membrane and surface of the biofilm from the bulk liquid, respectively (Fig. 1). The former is known as the co-diffusion biofilm and the latter is termed as the counter-diffusion biofilm^18^,^19^. This work aims to reveal the key difference of N_2_O production from the co- versus counter-diffusion nitrifying biofilms and the associated underlying mechanisms of N_2_O production by AOB from the co- and counter-diffusion biofilms using the two-pathway N_2_O^15^.

## Results

### The effect of co- and counter-diffusion on N_2_O production from AOB biofilms at varying biofilm thicknesses and oxygen loadings

Figure 2 illustrates the simulation results using the two-pathway model in both co- and counter-diffusion biofilms with different biofilm thicknesses and oxygen loading rates applied. Since there is no gas stripping process in bulk liquid, the effluent N_2_O concentration could be considered as the total N_2_O production by the AOB biofilm. For both diffusion geometries with biofilm thickness of 100 μm (Fig. 2A), the effluent N_2_O concentrations increased as oxygen loading increased initially, reached the peak at oxygen loading of approximately 4.5 m^3^/d and slightly decreased after oxygen loading further increased to 5.5 m^3^/d. N_2_O productions from the co-diffusion and counter-diffusion biofilms were similar at most oxygen loadings, except the ones between ~1.2–~2.8 m^3^/d, where N_2_O production from the co-diffusion biofilm was slightly higher than that from the counter-diffusion biofilm (Fig. 2A).

Figure 2B presents the effluent N_2_O concentration of the co- and counter-diffusion AOB biofilms with biofilm thickness of 200 μm. The maximum effluent N_2_O concentration was obtained at oxygen loading of approximately 4.25 m^3^/d for both co- and counter-diffusion biofilms. With the further increase or decrease of oxygen loading, a lower effluent N_2_O concentration was observed. The N_2_O production at biofilm thickness of 200 μm (Fig. 2B) was much higher than that from thinner biofilm (Fig. 2A). In the oxygen loading ranges of ~0.8–~2.4 m^3^/d and ~3.5–~5 m^3^/d, the N_2_O production from the co-diffusion biofilm was higher than that from counter-diffusion biofilm (Fig. 2B).

In Fig. 2C, for counter-diffusion biofilm, the increase of biofilm thickness from 200 to 300 μm didn’t alter the overall trend of effluent N_2_O concentration against increasing oxygen loading, but rather led to a minor elevated overall N_2_O production. However, the effluent N_2_O concentration in the co-diffusion biofilm with 300 μm biofilm thickness (Fig. 2C) peaked at oxygen loading of around 2.4 m^3^/d. By comparing N_2_O production from the co-diffusion biofilm with that from the counter-diffusion biofilm, it has been found: within oxygen loading of 0.24 to ~1 m^3^/d, the N_2_O productions from both diffusion geometries were similar to each other; in the range of ~1–~4 m^3^/d, the N_2_O production from the co-diffusion biofilm was substantially higher than that from the counter-diffusion biofilm; With the increase of the oxygen loading from ~4–~5.5 m^3^/d, the counter-diffusion biofilm produced more N_2_O (Fig. 2C).

To further identify the N_2_O source under the tested simulation conditions of Fig. 2C, the relative contributions of AOB denitrification pathway and NH_2_OH oxidation pathway to N_2_O production in AOB biofilm are illustrated in Figure S1 in Supplementary Material. For both diffusion geometries, the contribution of AOB denitrification pathway decreased with the increase of oxygen loading, accompanied by a corresponding increase of the contribution of NH_2_OH oxidation pathway. AOB denitrification pathway served to be the major contributor to N_2_O production (over 90% for all cases).

### Analysis of N_2_O production within the co- and counter- diffusion nitrifying biofilms at low oxygen loading (0.24 m^3^/d)

To reveal the mechanisms leading to the variation between N_2_O production from the co-diffusion biofilm and from the counter-diffusion biofilm, the simulation results of depth profiles with biofilm thickness of 300 μm at oxygen loadings of 0.24, 2.4 and 5.5 m^3^/d (see Fig. 2C) are presented in Figs 3, 4, 5.

In Fig. 3, with an influent ammonium concentration of 500 mg N/L, HRT of 12 hours and oxygen supply of 0.24 m^3^/d, the distribution of O_2_, NH_2_OH, Mox, Mred and N_2_O production rate via the two known pathways throughout the biofilm are depicted. The effluent concentration of NH_4_^+^ and 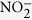 of the AOB biofilm for both diffusion geometries were ~470 and ~27 mg N/L, respectively as shown in Figure S2 in Supplementary Material. The concentration of NH_4_^+^ and 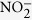 were relatively constant within the biofilm and close to the effluent concentrations (data not shown).

In the co-diffusion biofilm, the O_2_ concentration was ~0.055 mg O_2_/L at the outer layer of the biofilm, decreased gradually towards the base of biofilm and was depleted at a biofilm thickness of ~200 μm (Fig. 3A). The NH_2_OH concentration within the biofilm displayed a similar trend against biofilm depth and completely depleted at a biofilm depth of ~150 μm (Fig. 3A). The Mred concentration peaked at a biofilm thickness of ~200 μm, where the Mox concentration was minimal (Fig. 3B). The distribution of N_2_O production rate matches the predicted Mred stratification of the biofilm with negligible contribution from NH_2_OH oxidation pathway (Fig. 3C).

An opposite distribution profile is observed in the counter-diffusion biofilm. The concentration of O_2_ and NH_2_OH were maximal at the base of biofilm, decreased as biofilm thickness increased and was depleted at 100 μm and 150 μm, respectively (Fig. 3D). The highest point of Mred and lowest point of Mox were observed at a biofilm depth of 100 μm (Fig. 3E). The N_2_O production rate was also in good agreement with the Mred distribution profiles, which was mostly contributed by AOB denitrification pathway (Fig. 3F). The average concentrations of O_2_, NH_2_OH, Mred and Mox as well as the average N_2_O production rate, despite the varying distribution within the biofilm, were very similar in the co-diffusion and counter-diffusion biofilms, resulting in the similar N_2_O production in co- and counter diffusion biofilms under such low oxygen loading (Fig. 2C).

### Analysis of N_2_O production within the co- and counter- diffusion nitrifying biofilms at moderate oxygen loading (2.4 m^3^/d)

Figure 4 shows the stratification profiles of O_2_, NH_2_OH, Mox, Mred and N_2_O production rate via the two known pathways within the biofilms, with an influent ammonium concentration of 500 mg N/L, HRT of 12 hours and oxygen supply of 2.4 m^3^/d. The concentration of NH_4_^+^ and 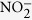 in effluent (Figure S2) and within the AOB biofilm for both diffusion geometries were in the ranges of ~222–~228 mg N/L and ~258–~260 mg N/L, respectively.

The distribution profiles of O_2_ and NH_2_OH in the co-diffusion biofilm were opposite to those in the counter-diffusion biofilm. The O_2_ and NH_2_OH within the biofilm decreased from the surface to the base and were depleted at 200 μm and 150 μm, respectively in the co-diffusion biofilm, while their concentrations decreased from base to surface and were depleted at 125 μm and 150 μm, respectively in the counter-diffusion biofilm (Fig. 4A,D). The co-diffusion biofilm has a lower maximum oxygen concentration, but a higher maximum NH_2_OH concentration in comparison to those in the counter-diffusion biofilm.

The Mred (/Mox) concentration peaked (/bottomed) at a biofilm depth of ~175 μm in the co-diffusion biofilm and at a biofilm depth of ~150 μm in the counter diffusion biofilm (Fig. 4B,E). The distribution of N_2_O production rate matched the predicted Mred stratification of the biofilm with major contribution from AOB denitrification pathway in both diffusion geometries (Fig. 4C,F). However, the maximum N_2_O production rate (~45 mg N/L/hour) in the co-diffusion biofilm was substantially higher than the one (~29 mg N/L/hour) in the counter-diffusion biofilm.

### Analysis of N_2_O production within the co- and counter- diffusion nitrifying biofilms at high oxygen loading (5.5 m^3^/d)

Figure 5 shows the distribution profiles of O_2_, NH_2_OH, Mox, Mred and N_2_O production rate via the two known pathways within the biofilm, with an influent ammonium concentration of 500 mg N/L, HRT of 12 hours and oxygen supply of 5.5 m^3^/d. Almost all of the influent ammonium were converted to nitrite under the high oxygen loading conditions (Figure S2).

The trend of O_2_ and NH_2_OH concentration against biofilm depth in both co- and counter-diffusion biofilms were in line with those at lower oxygen loadings in Figs 3 and 4 (Fig. 5A,D). The oxygen was exhausted at ~60 μm in the co-diffusion biofilm and at ~190 μm in the counter-diffusion biofilm (Fig. 5A–D). The NH_2_OH concentration was exhausted at the base of the co-diffusion biofilm and at a biofilm depth of 275 μm in the counter-diffusion biofilm.

The Mred (/Mox) concentration peaked (/bottomed) at a biofilm depth of ~275 μm in the co-diffusion biofilm (Fig. 5B,E). However, there were two highest points for Mred within the counter-diffusion biofilm: one at the base of biofilm and the other at the biofilm depth of ~290 μm, adjacent to the surface of biofilm. The biofilm distribution of Mred shaped the N_2_O production rate within the two types of biofilms (Fig. 5C,F). The AOB denitrification pathway was the main contributor throughout the biofilm, whereas a small contribution of the NH_2_OH oxidation pathway to N_2_O production occurred at the surface of the co-diffusion biofilm and at the base of the counter-diffusion biofilm.

### Model validation using experimental data

The proposed biofilm N_2_O model was tested using experimental data from a sequencing batch biofilm reactor (SBBR) performing partial nitritation^20^ The SSBR had a working volume of 6 L with plastic bio-carriers to facilitate biofilm formation. In each 12-h cycle, 3 L of synthetic wastewater containing 500 mg N/L of ammonium was fed into the reactor, resulting a hydraulic retention time of 24 hour. Off-gas N_2_O was collected in gas sampling bags at intervals of 30 min and analyzed by a gas chromatography. The experimental results of N_2_O emission from a typical cycle at steady state were used for model evaluation (Fig. 6). Intermittent aeration was applied and thus DO level in bulk liquid ranged from 0 to 1.5 mg O_2_/L for most cases. NH_4_^+^ concentration decreased from ~370 mg N/L to ~230 mg N/L, while 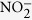 accumulated from ~120 mg N/L to ~260 mg N/L. The total emitted N_2_O in this cycle from SBBR is around 22 mg N, leading to an emission factor (defined by the ratio of emitted N-N_2_O and N-NH_4_^+^ loading) of 1.36%. The biofilm N_2_O model could satisfactorily capture the dynamics in terms of nitrogen conversions and N_2_O production (Fig. 6). The predicted N_2_O emission factor was around 1.3%, which well matched the measured value. The good agreement between model predictions and experimental data verified the validity and applicability of the N_2_O model for describing N_2_O production in nitrifying biofilm systems.

## Discussion

Although N_2_O production in suspended-growth sludge systems was extensively studied, little effort has been dedicated to investigating N_2_O production in biofilm systems, which are of growing significance during wastewater treatment. One of the biggest differences between the complete mixed compartment and the biofilm compartment is that the diffusion of substrates within the biofilm will result in stratified variations of the substrate concentrations. It is known that N_2_O would be affected by the precursors of ammonia oxidation^9^,^11^,^17^. With the development of MABR system, the variation of N_2_O between the conventional co-diffusion biofilm and the novel counter-diffusion biofilm is also expected due to the changed substrate distribution. A systematic experimental evaluation would take extremely a long time since each process condition would have to be tested for 6–12 months to be representative (when the biofilm system reaches steady state). This is likely the reason why not much data had been produced experimentally to date on such systems. Under such circumstances, this work used a previously-proposed and validated N_2_O model incorporating both AOB denitrification pathway and NH_2_OH oxidation pathway to give first insights into the difference of N_2_O productions from the co- and counter-diffusion biofilm systems.

Our results demonstrate that the stratified oxygen and NH_2_OH concentrations within the biofilm affect the N_2_O productions via AOB denitrification pathway and hydroxylamine oxidation pathway differently. For both diffusion geometries (Figs 3, 4, 5), AOB denitrification pathway is dominant over NH_2_OH oxidation pathway within the biofilm under the conditions of varying biofilm depths and oxygen loadings, which is consistent with the observations in a sequencing batch biofilm reactor^20^ as well as in growth-suspended nitrifying sludges^7^,^13^. A small contribution (less than 10%) of NH_2_OH oxidation pathway to total N_2_O production is observed at the outer layer of the co-diffusion biofilm and at the inner layer of the counter-diffusion biofilm, where the oxygen is more available. Its fraction decreases almost linearly upon the gradual depletion of oxygen through the biofilm (Figs 3, 4, 5), suggesting that oxygen may govern the N_2_O production via NH_2_OH oxidation pathway by regulating the electron supply.^13^ The AOB denitrification pathway in both co- and counter-diffusion biofilms is largely stimulated by the diffusion of NH_2_OH into the anoxic region of the biofilm, where no oxygen would compete for the electrons generated by NH_2_OH oxidation with 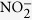 (Figs 3, 4, 5). High N_2_O production rates in the presence of NH_2_OH and 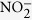 were also observed under anoxic conditions using suspended cultures^21^,^22^. The thicker biofilm generates higher N_2_O production is partly due to the extensive anoxic region for nitrite reduction (Fig. 2).

The simulation results indicate that the N_2_O production from co-diffusion biofilm is substantially higher than that from counter-diffusion biofilm at thick biofilm (300 μm) in the oxygen loading range of ~1–~4 m^3^/d and for other oxygen loadings the two have comparable effluent N_2_O concentrations (Fig. 2). This observation is attributed to the effect of co-diffusion and counter-diffusion geometry on substrate distribution. At low oxygen loading (0.24 m^3^/d), the distribution profiles of O_2_, NH_2_OH, Mred, Mox and N_2_O production rate via the two pathways in the co- and counter-diffusion biofilm are approximately mirror-symmetrical, leading to similar effluent N_2_O concentration (Fig. 3). However, at moderate oxygen loading (2.4 m^3^/d), the depth profiles of O_2_ and NH_2_OH are quite different in the two diffusion geometries. The co-diffusion biofilm involves a higher NH_2_OH concentration and lower O_2_ concentration at the surface of biofilm comparing to those at the base of the counter-diffusion biofilm (Fig. 4A,D). Consequently, the anoxic region with the presence of NH_2_OH and 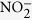 in co-diffusion is more extended than that in counter-diffusion biofilm, causing a higher peak of Mred and thus a higher N_2_O production from nitrite reduction (Fig. 4). By comparing the diffusion of NH_2_OH and oxygen within biofilm in Fig. 5A,D, it is seen that i) there is ~50 μm of anoxic region in co-diffusion biofilm (Fig. 5A), whereas the anoxic region in counter-diffusion biofilm is around 100 μm; ii) the NH_2_OH accumulation in anoxic region in co-diffusion biofilm is lower than that in counter-diffusion biofilm. Hence, for high oxygen loading (5.5 m^3^/d), N_2_O production from co-diffusion biofilm is slightly lower than that from counter-diffusion biofilm (Fig. 2C) due to the shrunken anoxic region with lower build-up of NH_2_OH in the co-diffusion biofilm.

The biofilm system with the combination of partial nitritation and Anammox has been widely studied and applied in treating digester liquor due to its zero requirement for organic carbon supplement, low sludge yield and low aeration energy consumption^23^,^24^,^25^,^26^. The two geometries of AOB biofilms, evaluated in this study, could be considered as the partial nitritation system producing nitrite for Anammox bacteria. A lower oxygen supply may hamper the autotrophic nitrogen removal efficiency owing to the insufficient nitrite for Anammox (Figure S2). A high oxygen supply (oxygen concentration > 1.0 mg O_2_/L) could exert an inhibitory effect on Anammox bacteria^26^. At moderate oxygen loading of 2.4 m^3^/d, both co- and counter-diffusion biofilms are able to provide a effluent 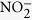/NH_4_^+^ ratio of 1.16 (Figure S2), which is very close to the theoretical stoichiometry for Anammox (1.32)^27^. Under such conditions, the counter-diffusion biofilm (e.g. MABR) should be preferably applied, rather than the conventional co-diffusion biofilm to reduce the N_2_O emissions from the system based on the simulation results of this study (Fig. 2). And for both co and counter-diffusion biofilm systems, the simulation results predict a lower N_2_O production at thinner biofilm thickness and lower bulk oxygen level (Fig. 2A) due to shrunken anoxic region and a decrease of NH_2_OH accumulation within the biofilm. Hence, a proper control of biofilm thickness by shear force and oxygen loading by aeration would help to mitigate N_2_O emission from such biofilm systems.

We further tested the biofilm model with low-strength ammonium in the influent (80 mg N/L), HRT of 6 hours and biofilm thickness of 300 μm. Figure S3 shows the effluent N_2_O concentration from co-diffusion and counter-diffusion biofilms under varying oxygen loadings. It is revealed that N_2_O productions from the co- and counter-diffusion biofilms are comparable to each other. The co-diffusion biofilm produces a little higher N_2_O at oxygen loading rate of around 1 m^3^/d, while counter-diffusion biofilm generates a slightly more N_2_O at oxygen loading of approximately 2 m^3^/d. Hence, for treating low-strength ammonium, N_2_O production is negligibly influenced by the diffusion geometry of the biofilm.

In order to fully clarify the interactions among N_2_O production, NH_2_OH and oxygen diffusion, and electron transportation by AOB in biofilms, the simulations in this work are performed based on the assumption that AOB are evenly distributed throughout the biofilm. It has been reported that AOB are able to grow on NH_2_OH oxidation and thus occupy an extended biofilm region^21^,^28^, while NOB are inhibited in the presence of NH_2_OH^29^,^30^. Nevertheless, further investigation is required to reveal the effect of diffusion geometry on N_2_O formation in more complex biofilms, where AOB, NOB, Anammox bacteria and/or heterotrophic organisms may co-exist and compete for substrates and growth space within the biofilm.

The model predictions of this work remain to be verified. However, the preliminary results will already support reactor operations under both steady-state and dynamic conditions. For instance, the simulation results can be used for process evaluation and directing pilot-plant research on application of membrane technology with special attention on N_2_O production. There is no doubt that the simulation-based observations are subject to further improvement, but the presented findings will serve as an excellent basis for future system development and optimization.

In summary, a previously established and validated two-pathway N_2_O model incorporating both AOB denitrification pathway and NH_2_OH oxidation pathway is used to assess the effect of co- and counter-diffusion on N_2_O production in nitrifying biofilms. AOB denitrification pathway is dominant over NH_2_OH oxidation pathway within the co- and counter-diffusion biofilms. The diffusion of NH_2_OH and oxygen within the biofilms forming anoxic layers with the presence of NH_2_OH and NO_2_^−^ result in a high N_2_O production rate via AOB denitrification pathway. The co-diffusion biofilm may generate substantially higher N_2_O than the counter-diffusion biofilm due to the higher accumulation of NH_2_OH in co-diffusion biofilm, especially under the condition of high-strength ammonium influent (e.g., 500 mg N/L), thick biofilm depth (e.g., 300 μm) and moderate oxygen loading (e.g., ~1–~4 m^3^/d). The effect of co- and counter-diffusion on N_2_O production and the related pathways from the nitrifying biofilms is minimal when treating low-strength nitrogenous wastewater (e.g., 80 mg N/L).

## Materials and Methods

### The co- and counter- diffusion biofilm reactor model

**Figure 1:**
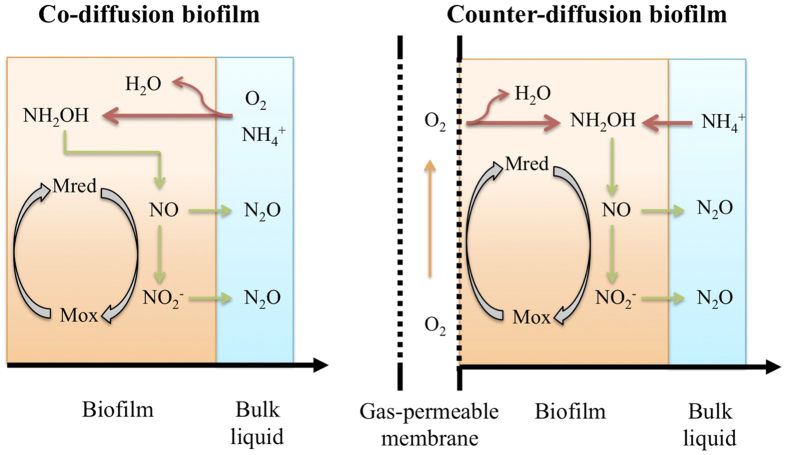
A simplified scheme of the co- (left) and counter-diffusion (right) biofilm structure with the two different N_2_O production pathways by AOB in nitrifying biofilms.

**Figure 2:**
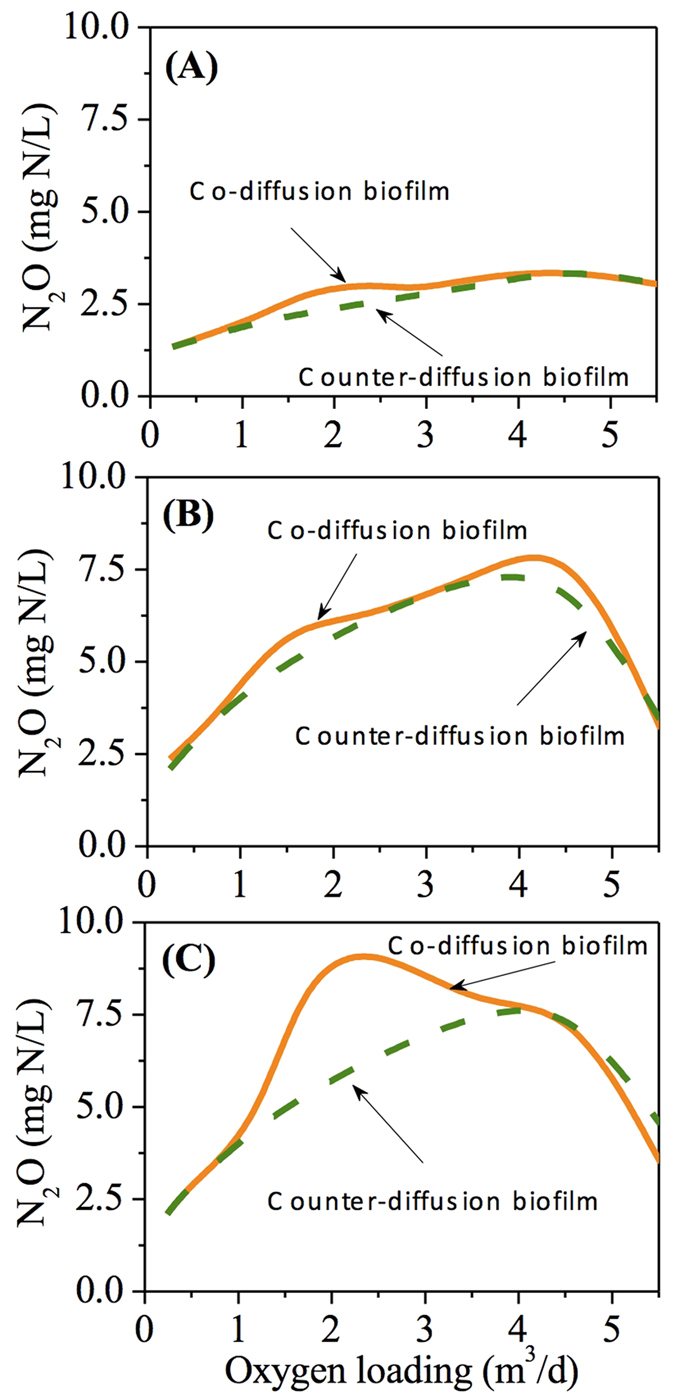
The effluent N_2_O concentrations from co- and counter-diffusion biofilm reactors as a function of oxygen loading with varying biofilm thicknesses applied: (**A**) 100 μm; (**B**) 200 μm; and (**C**) 300 μm. (The applied influent ammonium concentration and HRT are 500 mg N/L and 12 h, respectively).

**Figure 3:**
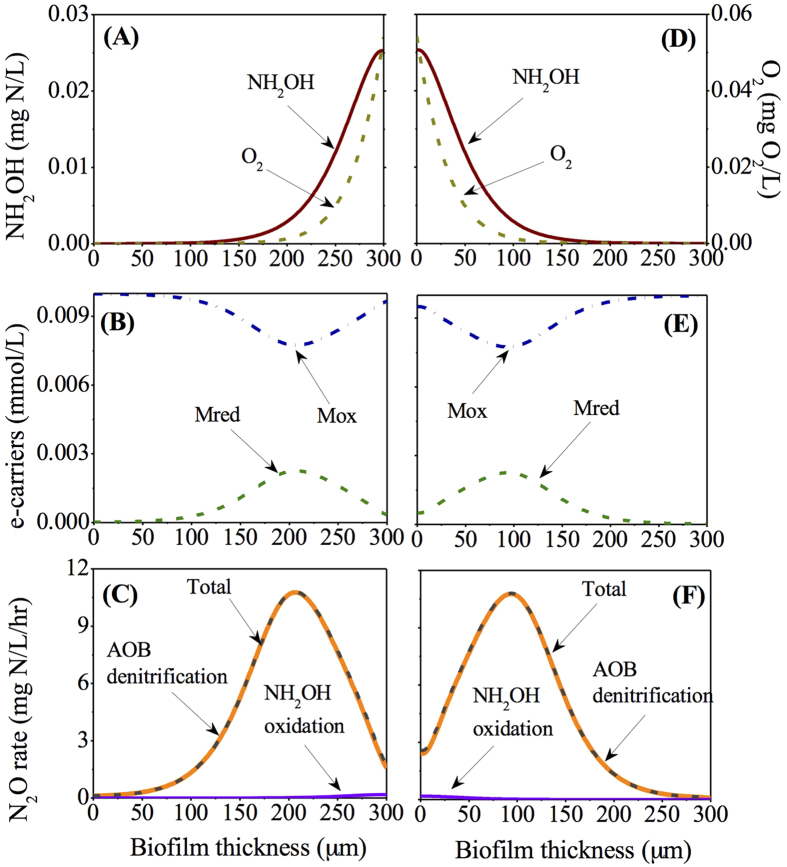
The depth profiles of NH_2_OH, O_2_, Mred, Mox and N_2_O production rates at low oxygen loading (0.24 m^3^/d) within the co-diffusion biofilm (**A–C**) and counter-diffusion biofilm (**D–F**).

**Figure 4:**
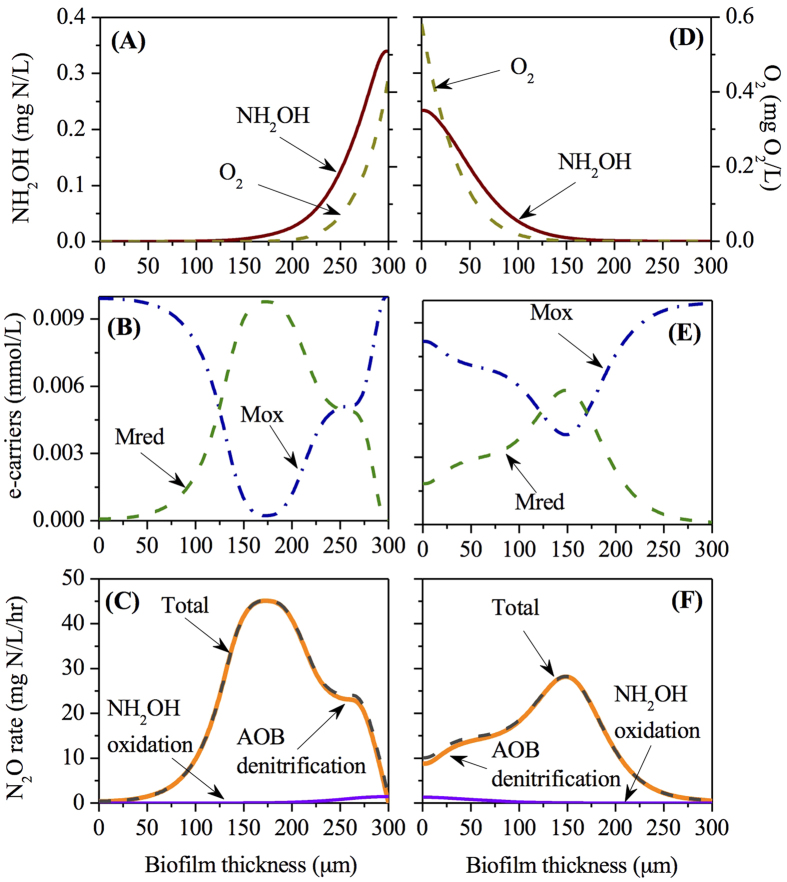
The depth profiles of NH_2_OH, O_2_, Mred, Mox and N_2_O production rates at moderate oxygen loading (2.4 m^3^/d) within the co-diffusion biofilm (**A–C**) and counter-diffusion biofilm (**D–F**).

**Figure 5:**
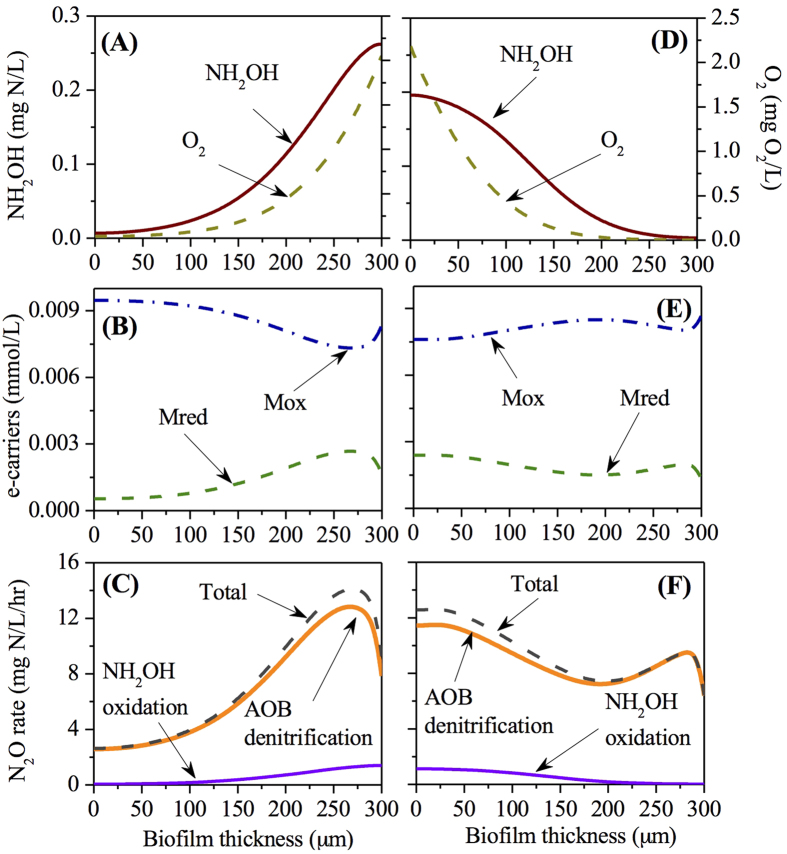
The depth profiles of NH_2_OH, O_2_, Mred, Mox and N_2_O production rates at high oxygen loading (5.5 m^3^/d) within the co-diffusion biofilm (**A–C**) and counter-diffusion biofilm (**D–F**).

**Figure 6:**
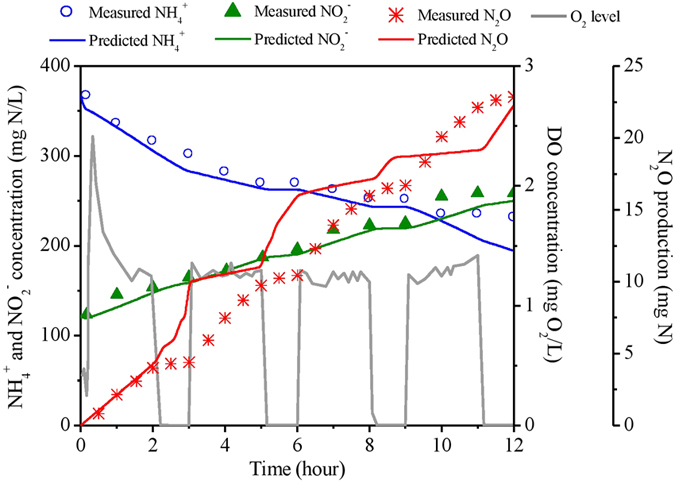
Comparison between model predictions and experimental data from a partial nitritation SBBR.

A one-dimensional AOB biofilm is constructed based on the assumption of a fixed biofilm thickness without any biomass growth, attachment or detachment and a uniform distribution of AOB throughout the biofilm for all cases, using software AQUASIM V2.1^31^. As shown in Fig. 1, the co-diffusion and counter-diffusion biofilm geometries are compared in the model: in the co-diffusion biofilm both oxygen and ammonium (NH_4_^+^) are supplied into the biofilm from the bulk liquid, whereas in the counter diffusion biofilm, the oxygen and NH_4_^+^ are dividedly provided to the biofilm base through a gas-permeable membrane and surface of the biofilm from the bulk liquid, respectively (Fig. 1). The biofilm reactor is modeled through consisting of a completely mixed gas compartment and a biofilm compartment (containing biofilm and bulk liquid). The gas compartment is linked to the bulk liquid in the co-diffusion biofilm and connected to the base of biofilm in the counter-diffusion biofilm through diffusive links. The oxygen concentration in the gas compartment is dependent on the gas flow rate and the applied gas pressure. The following kinetics (Equation 1) is used to model the flux of oxygen (*Fluxo*_*2*_) from the gas to the biofilm matrix compartment through the membrane in both biofilm reactors:


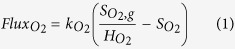


where *So*_2*,g*_ and *So*_2_ are the concentrations of oxygen in the gas and biofilm matrix compartments (g m^−3^), respectively, *Ko*_2_ is the overall mass transfer coefficient of oxygen (0.312 m d^−1^)^18^ and *Ho*_*2*_ is the Henry coefficient for oxygen (mole O_2_ m^−3^ gas/mole O_2_ m^−3^ liquid).

The biofilm specific surface area is set at 125 m^2^/m^3^. The biofilm density is 50000 g COD m^−3^. The water fraction of the biofilm matrix is kept constant at 0.75. Parameters with regard to the mass transfer coefficients for the substrates (e.g. ammonium, nitrite, oxygen etc.) are adopted from Hao *et al*.^32^. The biofilm model assumes no transportation of mediators due to the fact that the electron carriers are immobilized in the stationary microbial phase^17^.

### The two-pathway N_2_O model by AOB

The N_2_O model developed and validated by Ni *et al*.^15^ integrated both AOB denitrification pathway and NH_2_OH oxidation pathway and synthesized relevant biochemical reactions in conversion of ammonia (NH_3_), NH_2_OH, 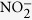, NO, N_2_O and O_2_. The electron transfer from oxidation to reduction was modeled by introducing electron mediators (Mred and Mox) as the new state variables. The definition and unit for these substrates are presented in Table S1 in Supplementary Material. The kinetics and stoichiometry of the model are shown in Table S2.

The N_2_O model is composed of seven processes (Table S2). In process 1, the oxidation of NH_3_ to NH_2_OH is accompanied by oxygen reduction with one O atom inserted into NH_2_OH and the other reduced to water (H_2_O). The conversion from Mred to Mox donates a pair of electrons to the O atom. Process 2 involves incomplete oxidation of NH_2_OH to NO, during which three electrons are generated. In process 3, the produced NO is then oxidized to 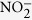, where one electron is formed. Mox would accept four electrons and be converted to Mred during oxidation of NH_2_OH to 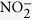. In process 4, the NO that is formed from incomplete NH_2_OH oxidation would be reduced to N_2_O (NH_2_OH oxidation pathway). Process 5 involves O_2_ reduction to H_2_O. In process 6, N_2_O is produced as the end product during nitrite reduction, which is described as a one-step process without NO intermediate to avoid NO and 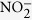 loop (AOB denitrification pathway). The Process 4, 5 and 6, accompanied by the conversion of Mred to Mox, would compete for the electrons generated from NH_2_OH oxidation and play important roles in regulating N_2_O production by AOB. In process 7, an increase of Mred is balanced by a decrease of Mox and vice versa (Mred 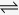 Mox + 2e^−^ + 2 H^+^), with the total level of electron carriers (C_tot_) being constant.

### Investigating the impacts of co- and counter- diffusion on N_2_O production

The N_2_O model by AOB used in this work has been evaluated and verified in both suspended-growth sludge systems^15^,^16^, and AOB biofilm system^17^. Hence we adapt those reported parameter values in literature (Table S3) to assess N_2_O production and other substrate conversions in biofilm system. This model-based evaluation approach has been well demonstrated in different microbial systems^17^,^32^,^33^,^34^,^35^.

The hydraulic retention time (HRT) for the biofilm compartment is set at 12 hours and the influent ammonium concentration is firstly kept constant at 500 mg N/L to investigate N_2_O production under high-strength ammonium conditions. Model simulations are then performed under varying conditions (biofilm thickness from 100–300 μm and oxygen loading rate from 0.24–5.5 m^3^/d) to provide insight into the effects of co- and counter diffusion on N_2_O production from the AOB biofilm. The underlying mechanisms are analyzed with the produced depth profiles of substrate concentrations and reaction rates in the co- and counter-diffusion AOB biofilms. Additional simulations at low influent ammonium concentration (80 mg N/L) are also conduced with the detailed results in Supplementary Material.

## Additional Information

**How to cite this article**: Peng, L. *et al*. Nitrous Oxide Production in Co- Versus Counter-Diffusion Nitrifying Biofilms. *Sci. Rep.* **6**, 28880; doi: 10.1038/srep28880 (2016).

## Supplementary Material

Supplementary Information
